# Gene model for the ortholog of *Roc1a* in *Drosophila simulans*

**DOI:** 10.17912/micropub.biology.001102

**Published:** 2025-09-24

**Authors:** Megan E. Lawson, Jakob Christian, Ryan J. Dufur, Braden Wright, Justin R. DiAngelo, Lindsey J. Long, Chinmay P. Rele, Laura K Reed

**Affiliations:** 1 The University of Alabama, Tuscaloosa, AL USA; 2 Penn State University, PA USA; 3 Oklahoma Christian University, Edmond, OK USA

## Abstract

Gene model for the ortholog of *Regulator of cullins 1a* ( *Roc1a* ) in the *Drosophila simulans* May 2017 (Princeton ASM75419v2/DsimGB2) Genome Assembly (GenBank Accession: GCA_000754195.3). This ortholog was characterized as part of a developing dataset to study the evolution of the Insulin/insulin-like growth factor signaling pathway (IIS) across the genus *Drosophila* using the Genomics Education Partnership gene annotation protocol for Course-based Undergraduate Research Experiences.

**Figure 1. Genomic neighborhood and gene model for Roc1a in D. simulans:**
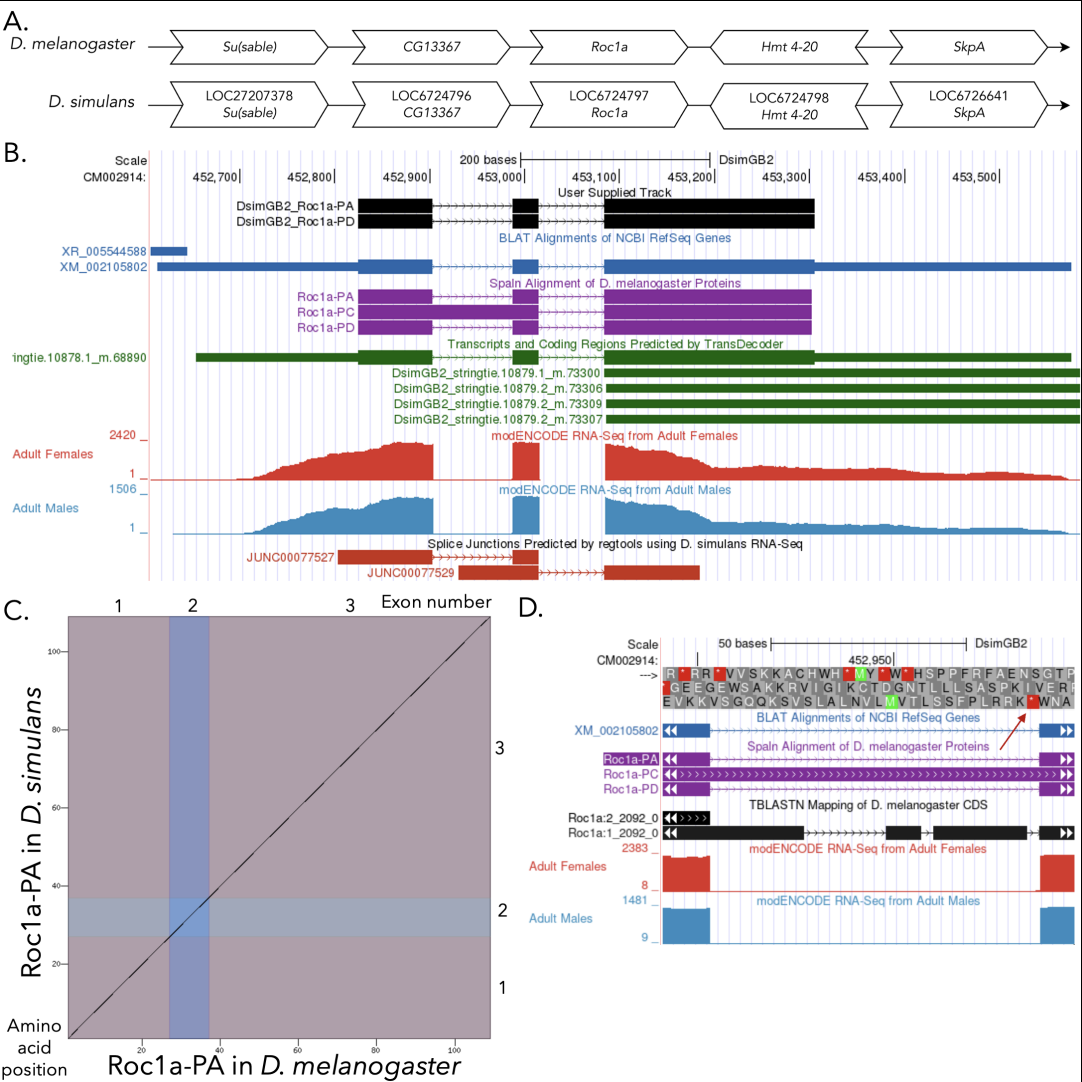
**
(A) Synteny comparison of the genomic neighborhoods for *Roc1a* in *Drosophila melanogaster* and *D.* *simulans* .** Thin underlying arrows indicate the DNA strand within which the target gene– *Roc1a* –is located in *D. melanogaster* (top) and *D. simulans* (bottom). The thin arrows pointing to the right indicate that *Roc1a* is on the positive (+) strand in *D. melanogaster* and *D. simulans.* The wide gene arrows pointing in the same direction as *Roc1a* are on the same strand relative to the thin underlying arrows, while wide gene arrows pointing in the opposite direction of *Roc1a* are on the opposite strand relative to the thin underlying arrows. White gene arrows in *D. simulans* indicate orthology to the corresponding gene in *D. melanogaster* . Gene symbols given in the *D. simulans* gene arrows indicate the orthologous gene in *D. melanogaster* , while the locus identifiers are specific to *D. simulans* . **(B) Gene Model in GEP UCSC Track Data Hub** (Raney et al., 2014). The coding-regions of *Roc1a* in *D. simulans* are displayed in the User Supplied Track (black); CDSs are depicted by thick rectangles and introns by thin lines with arrows indicating the direction of transcription. Subsequent evidence tracks include BLAT Alignments of NCBI RefSeq Genes (dark blue, alignment of Ref-Seq genes for *D. simulans* ), Spaln of *D. melanogaster* Proteins (purple, alignment of Ref-Seq proteins from *D. melanogaster* ), Transcripts and Coding Regions Predicted by TransDecoder (dark green), RNA-Seq from Adult Females and Adult Males (red and light blue, respectively; alignment of Illumina RNA-Seq reads from *D. simulans* ), and Splice Junctions Predicted by regtools using *D. simulans* RNA-Seq (SRP006203). Splice junctions shown have a minimum read-depth of 50 with >1000 supporting reads shown in red. **(C) Dot Plot of Roc1a-PA in *D. melanogaster* ( *x* -axis) vs. the orthologous peptide in *D. simulans* ( *y* -axis).** Amino acid number is indicated along the left and bottom; CDS (coding exon) number is indicated along the top and right, and CDSs are also highlighted with alternating colors. **(D) No open reading frame for hypothetical Roc1a-RC in *D. simulans* .** Coding frames for intron between first and second CDS for Roc1a-PA in UCSC Genome Browser. If an ortholog to Roc1a-RC was present, frame +3 should be open across the intron between the first two CDSs of the Roc1a-RA isoform. However, there is an in-frame stop codon (red arrow) indicating that the Roc1a-PC isoform likely does not exist in *D. simulans* . CDSs are depicted by thick rectangles and introns by thin lines with arrows indicating the direction of transcription. Subsequent evidence tracks include BLAT Alignments of NCBI RefSeq Genes (dark blue, alignment of Ref-Seq genes for *D. simulans* ), Spaln of *D. melanogaster* Proteins (purple, alignment of Ref-Seq proteins from *D. melanogaster* ), TBLASTN Mapping of *D. melanogaster* CDS (black), RNA-Seq from Adult Females and Adult Males (red and light blue, respectively; alignment of Illumina RNA-Seq reads from *D. simulans;* Gravely et al. 2011; SRP006203).

## Description

**Table:** 

* This article reports a predicted gene model generated by undergraduate work using a structured gene model annotation protocol defined by the Genomics Education Partnership (GEP; thegep.org ) for Course-based Undergraduate Research Experience (CURE). The following information in this box may be repeated in other articles submitted by participants using the same GEP CURE protocol for annotating Drosophila species orthologs of Drosophila melanogaster genes in the insulin signaling pathway. * "In this GEP CURE protocol students use web-based tools to manually annotate genes in non-model *Drosophila* species based on orthology to genes in the well-annotated model organism fruitfly *Drosophila melanogaster* . The GEP uses web-based tools to allow undergraduates to participate in course-based research by generating manual annotations of genes in non-model species (Rele et al., 2023). Computational-based gene predictions in any organism are often improved by careful manual annotation and curation, allowing for more accurate analyses of gene and genome evolution (Mudge and Harrow 2016; Tello-Ruiz et al., 2019). These models of orthologous genes across species, such as the one presented here, then provide a reliable basis for further evolutionary genomic analyses when made available to the scientific community.” (Myers et al., 2024). “The particular gene ortholog described here was characterized as part of a developing dataset to study the evolution of the Insulin/insulin-like growth factor signaling pathway (IIS) across the genus *Drosophila* . The Insulin/insulin-like growth factor signaling pathway (IIS) is a highly conserved signaling pathway in animals and is central to mediating organismal responses to nutrients (Hietakangas and Cohen 2009; Grewal 2009).” (Myers et al., 2024). “ *Roc1a* (also known as *Rbx1* ) is a member of the SCF E3 ubiquitin ligase complex and was originally identified through sequence similarity with vertebrate and yeast homologs and biochemical interaction studies (Bocca et al., 2001). *Roc1a* deletion mutants are lethal between the first and second larval instars and *Roc1a* mutant clones in imaginal discs have cell proliferation defects (Noureddine et al., 2002). In addition, Roc1a and other members of the SCF E3 ubiquitin ligase complex function in the pruning of larval neurons by targeting the insulin-responsive kinase Akt for ubiquitination and degradation, thus inhibiting insulin signaling (Wong et al., 2013).” (Lawson, 2025). “ *D. simulans (* NCBI:txid7237) is part of the *melanogaster* species group within the subgenus *Sophophora* of the genus *Drosophila* (Sturtevant 1939; Bock and Wheeler 1972) *.* It was first described by Sturtevant (1919). *D. simulans* is a sibling species to *D. melanogaster* , thus extensively studied in the context of speciation genetics and evolutionary ecology (Powell 1997). Historically, *D. simulans* was a tropical species native to sub-Saharan Africa (Lemeunier et al., 1986) where figs served as a primary host (Lachaise and Tsacas 1983). However, *D. simulans's* range has expanded worldwide within the last century as a human commensal using a broad range of rotting fruits as breeding sites (https://www.taxodros.uzh.ch, accessed 1 Feb 2023).” (Lawson et al., 2024).

We propose a gene model for the *D. simulans* ortholog of the *D. melanogaster* *Regulator of cullins 1a* ( *Roc1a* ) gene. The genomic region of the ortholog corresponds to the uncharacterized protein LOC6724797 (RefSeq accession XP_002105838.1 ) in the May 2017 (Princeton ASM75419v2/DsimGB2) Genome Assembly of *D. simulans* (GenBank Accession: GCA_000754195.3 ). This model is based on RNA-Seq data from *D. simulans* ( SRP006203 ; Graveley et al., 2011 *)* and *Roc1a* in *D. melanogaster* using FlyBase release FB2023_02 ( GCA_000001215.4 ; Larkin et al., 2021; Gramates et al., 2022; Jenkins et al., 2022).

***Synteny***

The reference gene, *Roc1a ,* occurs on chromosome X in *D. melanogaster* and is flanked upstream by *CG13367* and *suppressor of sable* ( *Su(sable)* ) and downstream by *Histone methyltransferase 4-20* ( *Hmt4-20* ) and *SKP1-related A* *( SkpA )* . The *tblastn* search of *D. melanogaster* Roc1a-PA (query) against the *D. simulans* (GenBank Accession: GCA_000754195.3 ) Genome Assembly (database) placed the putative ortholog of *Roc1a* within scaffold CM002914.1 at locus LOC6724797 ( XP_002105838.1 ) — with an E-value of 5e-61 and a percent identity of 67.92%. The putative ortholog is flanked upstream by LOC6724796 ( XP_002105837.1 ) and LOC27207378 ( XP_016037576.1 ), which correspond to *CG13367* and *Su(sable)* in *D. melanogaster* (E-value: 0.0 and 0.0; identity: 92.98% and 92.50%, respectively, as determined by *blastp* ) ( Figure 1A ; Altschul et al., 1990). The putative ortholog of *Roc1a* is flanked downstream by LOC6724798 ( XP_016037581.1 ) and LOC6726641 ( XP_016037582.1 ), which correspond to *Hmt4-20* and *SkpA* in *D. melanogaster* (E-value: 0.0 and 2e-118; identity: 98.65% and 100.00%, respectively, as determined by *blastp* ) ( Figure 1A ). The putative ortholog assignment for *Roc1a* in *D. simulans* is supported by the conservation of the localized gene order across both species.

***Protein Model***

*Roc1a* in *D. simulans* has one protein-coding isoform encoded by mRNA isoforms *Roc1a-RA* and *Roc1a-RD,* which differ in their UTRs and contain three CDSs ( Figure 1B ). Relative to the ortholog in *D. melanogaster* , the CDS number is conserved for these isoforms. However, *D. melanogaster* also has a third isoform with two CDSs, *Roc1a-RC* (Roc1a-PC), that is not present in *D. simulans* (see: “special characteristics”). The sequence of Roc1-PA in *D. simulans* has 100.00% identity (E-value: 8e-79) with Roc1a-PA in *D. melanogaster* , as determined by *blastp* ( Figure 1C ). Coordinates of this curated gene model for Roc1a-PA and Roc1a-PD are stored by NCBI at GenBank/BankIt (accession **BK064481 and BK064482** ). These data are also archived in the CaltechDATA repository (see “Extended Data” section below).

***Special characteristics of the protein model***

**Absence of Roc1a-PC in *D. simulans***

In *D. melanogaster* *Roc1a-RA* and *Roc1a-RD* have three CDSs whereas *Roc1a-RC* has two CDSs, with its first CDS (FlyBase ID: 1_2094_0) spanning the length of the first two CDSs of *Roc1a-RA* and *Roc1a-RD* (FlyBase IDs: 2_2094_0 and 3_2094_0) combined. All CDS IDs are based on FlyBase release FB2023_02; GCA_000001215.4 (Larkin et al., 2021; Gramates et al., 2022; Jenkins et al., 2022). In *D. melanogaster* , there are no in-frame stop codons in the longer first CDS of *Roc1a-RC* . However, in *D. simulans,* there is an in-frame stop codon present in what would otherwise be the first CDS of *Roc1a-RC,* which would prevent viable translation. This has been highlighted in Figure 1D indicating the presence of an in-frame stop codon in frame +3. Two other species more closely related to *D. similulans* than *D. melanogaster* ( *D. mauritiana* and *D. sechellia* ) also have a premature stop codon and identical nucleotide sequences in the same relative location in the hypothetical isoform *Roc1a-RC* , suggesting this characteristic of lacking a viable *Roc1a-RC* isoform is evolutionarily conserved within that species cluster, and is not the result of sequencing error. Further, the very low levels of RNA-Seq data supporting the expression of *Roc1a-RC* , in *D. simulans* suggests that Roc1a-PC is likely absent from *D. simulans.* Note, the extension of the left hand portion of splice junction JUNC00077529 prediction into the area of very low RNAseq signal is likely a by-product of the prediction algorithm that is not optimized for predicting splice sites from RNAseq reads that span two splice site as can occur with short exons (such as CDS2 in the *Roc1a-RA* and *Roc1a-RD* mRNAs). This special characteristic of *Roc1a-RC* is similar to the observations described in Lawson et al. (2025).

## Methods

Detailed methods including algorithms, database versions, and citations for the complete annotation process can be found in Rele et al. (2023). Briefly, students use the GEP instance of the UCSC Genome Browser v.435 ( https://gander.wustl.edu ; Kent WJ et al., 2002; Navarro Gonzalez et al., 2021) to examine the genomic neighborhood of their reference IIS gene in the *D. melanogaster* genome assembly (Aug. 2014; BDGP Release 6 + ISO1 MT/dm6). Students then retrieve the protein sequence for the *D. melanogaster* reference gene for a given isoform and run it using *tblastn* against their target *Drosophila* species genome assembly on the NCBI BLAST server ( https://blast.ncbi.nlm.nih.gov/Blast.cgi ; Altschul et al., 1990) to identify potential orthologs. To validate the potential ortholog, students compare the local genomic neighborhood of their potential ortholog with the genomic neighborhood of their reference gene in *D. melanogaster* . This local synteny analysis includes at minimum the two upstream and downstream genes relative to their putative ortholog. They also explore other sets of genomic evidence using multiple alignment tracks in the Genome Browser, including BLAT alignments of RefSeq Genes, Spaln alignment of *D. melanogaster* proteins, multiple gene prediction tracks (e.g., GeMoMa, Geneid, Augustus), and modENCODE RNA-Seq from the target species. Detailed explanation of how these lines of genomic evidenced are leveraged by students in gene model development are described in Rele et al. (2023). Genomic structure information (e.g., CDSs, intron-exon number and boundaries, number of isoforms) for the *D. melanogaster* reference gene is retrieved through the Gene Record Finder ( https://gander.wustl.edu/~wilson/dmelgenerecord/index.html ; Rele et al *.,* 2023). Approximate splice sites within the target gene are determined using *tblastn* using the CDSs from the *D. melanogaste* r reference gene. Coordinates of CDSs are then refined by examining aligned modENCODE RNA-Seq data, and by applying paradigms of molecular biology such as identifying canonical splice site sequences and ensuring the maintenance of an open reading frame across hypothesized splice sites. Students then confirm the biological validity of their target gene model using the Gene Model Checker ( https://gander.wustl.edu/~wilson/genechecker/index.html ; Rele et al., 2023), which compares the structure and translated sequence from their hypothesized target gene model against the *D. melanogaster* reference gene model. At least two independent models for a gene are generated by students under mentorship of their faculty course instructors. Those models are then reconciled by a third independent researcher mentored by the project leaders to produce the final model. Note: comparison of 5' and 3' UTR sequence information is not included in this GEP CURE protocol (Gruys et al., 2025).

## Data Availability

Description: Zip file containing FASTA, PEP, and GFF of the model. Resource Type: Model. DOI: https://doi.org/10.22002/67jye-ghd29

## References

[R1] Altschul SF, Gish W, Miller W, Myers EW, Lipman DJ (1990). Basic local alignment search tool.. J Mol Biol.

[R2] Bocca SN, Muzzopappa M, Silberstein S, Wappner P (2001). Occurrence of a putative SCF ubiquitin ligase complex in Drosophila.. Biochem Biophys Res Commun.

[R3] Bock, I.R., Wheeler, M.R. 1972. The Drosophila melanogaster species group. Univ. Texas Publs Stud. Genet. 7(7213): 1--102. FBrf0024428

[R4] Clark AG, Eisen MB, Smith DR, Bergman CM, Oliver B, Markow TA, Kaufman TC, Kellis M, Gelbart W, Iyer VN, Pollard DA, Sackton TB, Larracuente AM, Singh ND, Abad JP, Abt DN, Adryan B, Aguade M, Akashi H, Anderson WW, Aquadro CF, Ardell DH, Arguello R, Artieri CG, Barbash DA, Barker D, Barsanti P, Batterham P, Batzoglou S, Begun D, Bhutkar A, Blanco E, Bosak SA, Bradley RK, Brand AD, Brent MR, Brooks AN, Brown RH, Butlin RK, Caggese C, Calvi BR, Bernardo de Carvalho A, Caspi A, Castrezana S, Celniker SE, Chang JL, Chapple C, Chatterji S, Chinwalla A, Civetta A, Clifton SW, Comeron JM, Costello JC, Coyne JA, Daub J, David RG, Delcher AL, Delehaunty K, Do CB, Ebling H, Edwards K, Eickbush T, Evans JD, Filipski A, Findeiss S, Freyhult E, Fulton L, Fulton R, Garcia AC, Gardiner A, Garfield DA, Garvin BE, Gibson G, Gilbert D, Gnerre S, Godfrey J, Good R, Gotea V, Gravely B, Greenberg AJ, Griffiths-Jones S, Gross S, Guigo R, Gustafson EA, Haerty W, Hahn MW, Halligan DL, Halpern AL, Halter GM, Han MV, Heger A, Hillier L, Hinrichs AS, Holmes I, Hoskins RA, Hubisz MJ, Hultmark D, Huntley MA, Jaffe DB, Jagadeeshan S, Jeck WR, Johnson J, Jones CD, Jordan WC, Karpen GH, Kataoka E, Keightley PD, Kheradpour P, Kirkness EF, Koerich LB, Kristiansen K, Kudrna D, Kulathinal RJ, Kumar S, Kwok R, Lander E, Langley CH, Lapoint R, Lazzaro BP, Lee SJ, Levesque L, Li R, Lin CF, Lin MF, Lindblad-Toh K, Llopart A, Long M, Low L, Lozovsky E, Lu J, Luo M, Machado CA, Makalowski W, Marzo M, Matsuda M, Matzkin L, McAllister B, McBride CS, McKernan B, McKernan K, Mendez-Lago M, Minx P, Mollenhauer MU, Montooth K, Mount SM, Mu X, Myers E, Negre B, Newfeld S, Nielsen R, Noor MA, O'Grady P, Pachter L, Papaceit M, Parisi MJ, Parisi M, Parts L, Pedersen JS, Pesole G, Phillippy AM, Ponting CP, Pop M, Porcelli D, Powell JR, Prohaska S, Pruitt K, Puig M, Quesneville H, Ram KR, Rand D, Rasmussen MD, Reed LK, Reenan R, Reily A, Remington KA, Rieger TT, Ritchie MG, Robin C, Rogers YH, Rohde C, Rozas J, Rubenfield MJ, Ruiz A, Russo S, Salzberg SL, Sanchez-Gracia A, Saranga DJ, Sato H, Schaeffer SW, Schatz MC, Schlenke T, Schwartz R, Segarra C, Singh RS, Sirot L, Sirota M, Sisneros NB, Smith CD, Smith TF, Spieth J, Stage DE, Stark A, Stephan W, Strausberg RL, Strempel S, Sturgill D, Sutton G, Sutton GG, Tao W, Teichmann S, Tobari YN, Tomimura Y, Tsolas JM, Valente VL, Venter E, Venter JC, Vicario S, Vieira FG, Vilella AJ, Villasante A, Walenz B, Wang J, Wasserman M, Watts T, Wilson D, Wilson RK, Wing RA, Wolfner MF, Wong A, Wong GK, Wu CI, Wu G, Yamamoto D, Yang HP, Yang SP, Yorke JA, Yoshida K, Zdobnov E, Zhang P, Zhang Y, Zimin AV, Baldwin J, Abdouelleil A, Abdulkadir J, Abebe A, Abera B, Abreu J, Acer SC, Aftuck L, Alexander A, An P, Anderson E, Anderson S, Arachi H, Azer M, Bachantsang P, Barry A, Bayul T, Berlin A, Bessette D, Bloom T, Blye J, Boguslavskiy L, Bonnet C, Boukhgalter B, Bourzgui I, Brown A, Cahill P, Channer S, Cheshatsang Y, Chuda L, Citroen M, Collymore A, Cooke P, Costello M, D'Aco K, Daza R, De Haan G, DeGray S, DeMaso C, Dhargay N, Dooley K, Dooley E, Doricent M, Dorje P, Dorjee K, Dupes A, Elong R, Falk J, Farina A, Faro S, Ferguson D, Fisher S, Foley CD, Franke A, Friedrich D, Gadbois L, Gearin G, Gearin CR, Giannoukos G, Goode T, Graham J, Grandbois E, Grewal S, Gyaltsen K, Hafez N, Hagos B, Hall J, Henson C, Hollinger A, Honan T, Huard MD, Hughes L, Hurhula B, Husby ME, Kamat A, Kanga B, Kashin S, Khazanovich D, Kisner P, Lance K, Lara M, Lee W, Lennon N, Letendre F, LeVine R, Lipovsky A, Liu X, Liu J, Liu S, Lokyitsang T, Lokyitsang Y, Lubonja R, Lui A, MacDonald P, Magnisalis V, Maru K, Matthews C, McCusker W, McDonough S, Mehta T, Meldrim J, Meneus L, Mihai O, Mihalev A, Mihova T, Mittelman R, Mlenga V, Montmayeur A, Mulrain L, Navidi A, Naylor J, Negash T, Nguyen T, Nguyen N, Nicol R, Norbu C, Norbu N, Novod N, O'Neill B, Osman S, Markiewicz E, Oyono OL, Patti C, Phunkhang P, Pierre F, Priest M, Raghuraman S, Rege F, Reyes R, Rise C, Rogov P, Ross K, Ryan E, Settipalli S, Shea T, Sherpa N, Shi L, Shih D, Sparrow T, Spaulding J, Stalker J, Stange-Thomann N, Stavropoulos S, Stone C, Strader C, Tesfaye S, Thomson T, Thoulutsang Y, Thoulutsang D, Topham K, Topping I, Tsamla T, Vassiliev H, Vo A, Wangchuk T, Wangdi T, Weiand M, Wilkinson J, Wilson A, Yadav S, Young G, Yu Q, Zembek L, Zhong D, Zimmer A, Zwirko Z, Jaffe DB, Alvarez P, Brockman W, Butler J, Chin C, Gnerre S, Grabherr M, Kleber M, Mauceli E, MacCallum I, Drosophila 12 Genomes Consortium. (2007). Evolution of genes and genomes on the Drosophila phylogeny.. Nature.

[R5] Gramates LS, Agapite J, Attrill H, Calvi BR, Crosby MA, Dos Santos G, Goodman JL, Goutte-Gattat D, Jenkins VK, Kaufman T, Larkin A, Matthews BB, Millburn G, Strelets VB, the FlyBase Consortium. (2022). Fly Base: a guided tour of highlighted features.. Genetics.

[R6] Graveley BR, Brooks AN, Carlson JW, Duff MO, Landolin JM, Yang L, Artieri CG, van Baren MJ, Boley N, Booth BW, Brown JB, Cherbas L, Davis CA, Dobin A, Li R, Lin W, Malone JH, Mattiuzzo NR, Miller D, Sturgill D, Tuch BB, Zaleski C, Zhang D, Blanchette M, Dudoit S, Eads B, Green RE, Hammonds A, Jiang L, Kapranov P, Langton L, Perrimon N, Sandler JE, Wan KH, Willingham A, Zhang Y, Zou Y, Andrews J, Bickel PJ, Brenner SE, Brent MR, Cherbas P, Gingeras TR, Hoskins RA, Kaufman TC, Oliver B, Celniker SE (2010). The developmental transcriptome of Drosophila melanogaster.. Nature.

[R7] Grewal SS (2008). Insulin/TOR signaling in growth and homeostasis: a view from the fly world.. Int J Biochem Cell Biol.

[R8] Gruys ML, Sharp MA, Lill Z, Xiong C, Hark AT, Youngblom JJ, Rele CP, Reed LK (2025). Gene model for the ortholog of Glys in Drosophila simulans.. MicroPubl Biol.

[R9] Hietakangas V, Cohen SM (2009). Regulation of tissue growth through nutrient sensing.. Annu Rev Genet.

[R10] Jenkins VK, Larkin A, Thurmond J, FlyBase Consortium (2022). Using FlyBase: A Database of Drosophila Genes and Genetics.. Methods Mol Biol.

[R11] Kent WJ, Sugnet CW, Furey TS, Roskin KM, Pringle TH, Zahler AM, Haussler D (2002). The human genome browser at UCSC.. Genome Res.

[R12] Lachaise, D., Tsacas, L. 1983. Breeding-sites of tropical African Drosophilids. Ashburner, Carson, Thompson, 1981-1986 3d: 221--332. FBrf0038884

[R13] Larkin A, Marygold SJ, Antonazzo G, Attrill H, Dos Santos G, Garapati PV, Goodman JL, Gramates LS, Millburn G, Strelets VB, Tabone CJ, Thurmond J, FlyBase Consortium. (2021). FlyBase: updates to the Drosophila melanogaster knowledge base.. Nucleic Acids Res.

[R14] Lawson ME, Jones GM, Runion M, Sims D, Young A, Briggs O, Long LJ, Tin Chi Chak S, Bose I, Rele CP, Reed LK (2024). Gene model for the ortholog of ImpL2 in Drosophila simulans.. MicroPubl Biol.

[R15] Lawson ME, Wellik IG, Alvarado B, German T, Thompson JS, Long LJ, DiAngelo JR, Yang MA, Rele CP, Reed LK (2025). Gene model for the ortholog of Roc1a in Drosophila eugracilis.. MicroPubl Biol.

[R16] Lemeunier, F., David, J., Tsacas, L., Ashburner, M. 1986. The melanogaster species group. Ashburner, Carson, Thompson, 1981-1986 e: 147--256. FBrf0043749

[R17] Myers A, Hoffman A, Natysin M, Arsham AM, Stamm J, Thompson JS, Rele CP, Reed LK (2024). Gene model for the ortholog Myc in Drosophila ananassae.. MicroPubl Biol.

[R18] Mudge JM, Harrow J (2016). The state of play in higher eukaryote gene annotation.. Nat Rev Genet.

[R19] Navarro Gonzalez J, Zweig AS, Speir ML, Schmelter D, Rosenbloom KR, Raney BJ, Powell CC, Nassar LR, Maulding ND, Lee CM, Lee BT, Hinrichs AS, Fyfe AC, Fernandes JD, Diekhans M, Clawson H, Casper J, Benet-Pagès A, Barber GP, Haussler D, Kuhn RM, Haeussler M, Kent WJ (2021). The UCSC Genome Browser database: 2021 update.. Nucleic Acids Res.

[R20] Noureddine MA, Donaldson TD, Thacker SA, Duronio RJ (2002). Drosophila Roc1a encodes a RING-H2 protein with a unique function in processing the Hh signal transducer Ci by the SCF E3 ubiquitin ligase.. Dev Cell.

[R21] Pélandakis M, Solignac M (1993). Molecular phylogeny of Drosophila based on ribosomal RNA sequences.. J Mol Evol.

[R22] Powell JR. 1997. Progress and prospects in evolutionary biology: the Drosophila model. Oxford University Press, ISBN: 9780195076912

[R23] Raney BJ, Dreszer TR, Barber GP, Clawson H, Fujita PA, Wang T, Nguyen N, Paten B, Zweig AS, Karolchik D, Kent WJ (2013). Track data hubs enable visualization of user-defined genome-wide annotations on the UCSC Genome Browser.. Bioinformatics.

[R24] Rele Chinmay P., Sandlin Katie M., Leung Wilson, Reed Laura K. (2023). Manual annotation of Drosophila genes: a Genomics Education Partnership protocol. F1000Research.

[R25] Sturtevant AH (1939). On the Subdivision of the Genus Drosophila.. Proc Natl Acad Sci U S A.

[R26] Sturtevant, A.H., 1919. A new species closely resembling to Drosophila melanogaster. Psyche 26: 488–500. FBrf0000977

[R27] Tello-Ruiz MK, Marco CF, Hsu FM, Khangura RS, Qiao P, Sapkota S, Stitzer MC, Wasikowski R, Wu H, Zhan J, Chougule K, Barone LC, Ghiban C, Muna D, Olson AC, Wang L, Ware D, Micklos DA (2019). Double triage to identify poorly annotated genes in maize: The missing link in community curation.. PLoS One.

[R28] Wong JJ, Li S, Lim EK, Wang Y, Wang C, Zhang H, Kirilly D, Wu C, Liou YC, Wang H, Yu F (2013). A Cullin1-based SCF E3 ubiquitin ligase targets the InR/PI3K/TOR pathway to regulate neuronal pruning.. PLoS Biol.

